# Trailgazers: A Scoping Study of Footfall Sensors to Aid Tourist Trail Management in Ireland and Other Atlantic Areas of Europe

**DOI:** 10.3390/s21062038

**Published:** 2021-03-13

**Authors:** Kyle Madden, Elaine Ramsey, Sharon Loane, Joan Condell

**Affiliations:** 1 Department of Global Business and Enterprise, Ulster University, Magee Campus, Northland Rd, Londonderry BT48 7JL, UK; e.ramsey@ulster.ac.uk (E.R.); sp.loane@ulster.ac.uk (S.L.); 2 School of Computing, Engineering & Intel. Sys, Ulster University, Magee Campus, Northland Rd, Londonderry BT48 7JL, UK; j.condell@ulster.ac.uk

**Keywords:** footfall sensors, people counting, outdoor trails, tourism management, Atlantic Area

## Abstract

This paper examines the current state of the art of commercially available outdoor footfall sensor technologies and defines individually tailored solutions for the walking trails involved in an ongoing research project. Effective implementation of footfall sensors can facilitate quantitative analysis of user patterns, inform maintenance schedules and assist in achieving management objectives, such as identifying future user trends like cyclo-tourism. This paper is informed by primary research conducted for the EU funded project TrailGazersBid (hereafter referred to as TrailGazers), led by Donegal County Council, and has Sligo County Council and Causeway Coast and Glens Council (NI) among the 10 project partners. The project involves three trails in Ireland and five other trails from Europe for comparison. It incorporates the footfall capture and management experiences of trail management within the EU Atlantic area and desk-based research on current footfall technologies and data capture strategies. We have examined 6 individual types of sensor and discuss the advantages and disadvantages of each. We provide key learnings and insights that can help to inform trail managers on sensor options, along with a decision-making tool based on the key factors of the power source and mounting method. The research findings can also be applied to other outdoor footfall monitoring scenarios.

## 1. Introduction

The TrailGazers EU project is funded under the EU Interreg Atlantic Area (AA) Programme, with an overall value of €2.6 million and involves a consortium of 10 partners from various Atlantic Area Regions, including Republic of Ireland (ROI), France, Portugal, Spain, and the UK. The project is led by Donegal County Council, Ireland, and aims to develop a transnationally tested framework that will enable the future management and promotion of trails across the Atlantic Area in Europe. The framework will use digital technologies and indicators to develop trails in an environmentally friendly, sustainable, cost-effective and highly innovative manner. The Northern Ireland project partner (Higher Education institution) is responsible for the Trail Technology Deployment work package. Some of their key tasks include the specification and installation of footfall sensors along the eight walking trails in the respective partner regions. Each trail site requires a tailored technology solution to maximise footfall data accuracy; and the development of an open-source trail monitor/dashboard that links the captured sensor data. This data will be used as the basis of footfall data insights on trail usage patterns, and the prediction of future visitor quantities. A key expectation from the trails dashboard is to provide a one-stop-shop to assist with future planning and tourism management for project partners and invited stakeholders. All of the outcomes for the project will depend first and foremost on the ability to scope out and procure footfall sensors that will be able to capture footfall data along the trail sites, each with its own unique topography. The new technology solutions should complement existing sensor infrastructures where possible on trail sites.

The paper is organised as follows. Firstly, we discuss key elements around footfall data generated from sensors. We also examine how footfall data is currently used across the TrailGazers consortium for key areas of trail management, to gather practical ground-level insight to inform decisions and also to help improve trail management strategies. Outdoor sensor usage on trails is discussed. Thereafter the pilot trail sites are presented and their existing sensor technologies are reviewed; along with a decision tool for sensor technology selection. The paper culminates with a set of sensor technology recommendations for the pilot trails and final conclusion.

## 2. Background

‘Footfall’ is a time-series statistic that is used to measure the number of visitors or passers-by that are received within in a specific location and a given timeframe. Aggregated footfall data is used to compare the popularity of each point of installation; historical data can be gathered and stored over time and can be used to monitor an increase or decrease in visitors. Traditionally, footfall counts would be produced manually by an employee physically attending the location and counting each visitor at a certain point. This approach could be performed seasonally or even annually due to the cost of labour. Infrequent footfall counts can reduce the accuracy of predictions and forecasts due to the granularity of the data. Footfall counters are automatic, sensing technology-based approaches for quantifying footfall statistics. This technology is also often referred to as ‘people counters’ or ‘pedestrian counters’. There is a myriad of different sensor technology implementations aimed at detecting footfall, with differing levels of detection accuracy and distinction between objects. Popular technologies are discussed in more detail later in the paper. The evolution of footfall technology and readily available footfall sensors has made visitor analytics more attractive, particularly since footfall data insights can be combined with other Key Performance Indicators (KPIs), e.g., ratio of returning visitors.

### 2.1. Trail Management and Footfall Data

The authors in [1] comment, “Trails have helped form the basis of human mobility patterns and have been essential to travel and tourism”. Many old trail routes are still in use today, functioning both as important passageways and as tourist attractions [2]. Walking for recreation has become one of the most popular outdoor activities [3] providing easy access to the environment and nature [1], exercise [4] and also allows interaction with elements of cultural heritage [5]. This trend is well recognised in the Irish context, and increased activity tourism is a key pillar of the Government’s Action Plan for Rural Development [6]. Funding through the Outdoor Recreation Infrastructure Scheme is available in Ireland to walking trails, alongside other recreation infrastructure. This scheme has promoted growth and approximately 14 new trails have opened in Co. Sligo [7]. In Northern Ireland, the efficacy of trails for rural development has led to several large new projects, for example, Darkley Forest Trail and the Gilford River Trail. Funding sources include DEARA’s Targeting Rural Poverty and Social Isolation (TRPSI) programme and the National Lottery through Sport Northern Ireland’s ‘Every Body Active: Outdoor Spaces’ programme. 

Trail Related Tourism (TRT) is a significant factor in regional development considerations, given the potential social and economic benefits generated for residents and communities [3]. Therefore, destination management requires coordinated management of all the elements that make up a destination management plan. Such plans necessitate a strategic focus to manage trails, and allocate resources, ensure sustainability, protect environments and leverage economic development opportunities. The use of smart sensors to measure footfall on trails can support these activities. Additionally, footfall data and the intelligent forecasting or prediction of tourist numbers can be linked to future economic development around trailheads [8], and allow sustainable and environmentally appropriate trail-related tourism to take place [9], particularly, given the acceleration of trail construction in Ireland and the Atlantic Area of Europe in recent years.

The TrailGazers project seeks to address many of the issues highlighted above and will utilise both technology and (output) data to identify segments of trails that are facing environmental threats due to increased trail usage. Some of the TrailGazers pilot sites in Ireland and across the EU already have sensors in place to inform trail management. Partners’ trail data will be used for reporting utilisation figures to many different organisations, including government bodies, higher-level tourism boards, senior management, national parks, wildlife services and social development projects, publicity (PR) on local media, websites and tourism brochures. Some trail sites installed sensors at the beginning of a previous project initiative to monitor progress on targets achieved and increased visitor numbers. Some partners have policies around sharing data with local authorities. Moreover, the data is used to establish the best closure dates for maintenance, and to support grant applications for trail maintenance and future development or expansion. Prioritisation of maintenance budget allocation is an important use case, particularly if there are multiple local trails or routes which have a single point of management. Ultimately, the data is used to establish a long-term archive of visitor data and inform basic forecasts about upcoming seasons. 

### 2.2. TrailGazers Pilot Trail Sites

There are eight TrailGazers pilot trails located in the Atlantic Area (AA) in the project partner regions [10]. The selected trails across Ireland and the EU have varying characteristics and are at different stages of development but are a true reflection of the thematic and territorial dimensions of the AA region. These trails will be used for the development of tailored destination management plans that will stimulate economic development by harnessing the potential of trails natural and cultural assets. Each trail could present different challenges in capturing footfall, as the climate or conditions may not be consistent between these locations.

#### 2.2.1. Inch Levels, Donegal, Ireland

Inch Levels Wildfowl Reserve is situated on the eastern shores of Lough Swilly in County Donegal, Ireland. The Reserve is one of the most important wetlands in the country and has extensive feeding areas and safe resting and roosting sites for wintering waterfowl, notably swans and geese. It also houses breeding terns, gulls, waders and duck, and also provides a haven for farmland birds and birds of prey. Being on the westernmost part of Europe it also attracts a fair share of unusual birds throughout the year [11].

This trail has eight existing sensors—three are inductive loop sensors (used to count vehicles entering car parks) and the remaining five are Photoelectric Infra-Red (PIR) break-beam type sensors for detecting people entering certain areas of the trail. There are several possible entry and exit points which increases the difficulty in accurately detecting individual trail users; the same visitor could trigger multiple sensors throughout their journey, causing overlapped count figures and therefore inflated visitor numbers. Agresssive calibration may be required to reduce this this occurrence, which can then reduce vistor counts. Therefore, more accurate data could be valuable for this trial, as the quantitative findings that can currently be retrieved from existing sensors may not be as accurate.

#### 2.2.2. Knocknarea/Killspugbrone Loop, Sligo, Ireland

Knocknarea Mountain Sligo was formed from limestone over 300 million years ago and has been an important ritual focal point since Neolithic times. Along the Knocknarea/Killspugbrone Loop trail walkers can experience the coastal habitat, including sand dunes, salt marsh and pine woodland, as well as magnificent views out over Sligo Bay [12].

There are six uni-directional PIR Break Beam sensors installed amongst the Knocknarea and Killaspugbrone Trail Loop. These sensors require manual collection and offline data analysis, the software may be ‘deprecated’ and no longer actively supported. These sensors may need to be replaced, though it may be possible to integrate existing sensor data with the new dashboard.

#### 2.2.3. Vía Verde Del Plazaola, Navarra, Spain

The Vía Verde Del Plazaola Trail in Navarra, Spain, emerged out of an old railway track that once linked the cities of San Sebastián and Pamplona through a spectacular winding route. As a consequence of severe floods in 1953 and strong competition from the coach lines, the track was closed in 1958. More recently, 53 km of the track has been adapted as a greenway for cyclists and walkers [13]. 

The trail is noticeably long and has few points of entry and exit, which could help with increasing footfall count accuracy. There are two existing sensors—a dual inductive loop sensor to directionally count bicycles and a 3D Stereoscopic Camera to count people. Both sensors are placed in the same detection area.

#### 2.2.4. La Caldera De Taburiente, La Palma, Isla Canarias, Spain

The La Caldera de Taburiente trail passes through a pine forest that has a rich undergrowth of endemic species. The paths also give walkers access to some landmark sites which are the product of landslides caused by volcanic eruptions over the centuries. In addition, there are a multitude of streams and waterfalls to enjoy [14]. 

This is a circular trail with several different points of entry and exit. There are three existing ‘pressure slab’ type sensors to quantify footfall—these sensors detect footfall based on a weight being applied to a large ‘slab’ sensor buried under the ground. They can be susceptible to changes in environment, soil shifting from bad weather and even soil compaction from high levels of traffic (footfall) can reduce the effectiveness of this sensor type. 

#### 2.2.5. Sete Vales Suspensos, Algarve, Portugal

The Seven Hanging Valleys trail in the Algarve, Portugal. was recently voted the best hiking destination in Europe, allowing hikers and walkers to enjoy the landscapes of the Algarve coast. The trail is approximately 6 km (12 km round trip) long and connects Praia da Marinha and Vale de Centeanes Beach through a continuous line of cliffs, interspersed by waterways that, in winter, flow above sea level. This gives rise to ‘hanging valleys’ [15].

The trail is linear and has several trail ‘legs’ where users can enter and exit. This could cause difficulty in quantifying trail users as it may be very difficult to derive an overarching ‘Total Trail Users’ figure; visitors could be counted several times at different points of the trail and therefore the accuracy of a summative visitor ‘total count’ is not guaranteed. There are four bi-directional sensors and four non-directional sensors in place.

#### 2.2.6. Chemin De Mémoires, Ille-Et-Vilaine, France

Chemin De Mémoires (The Path of Memory(s) Ille-Et-Vilaine, France goes through 8 km of Louvigné du Désert, also known as the ‘Capital of Granite’. It is located in the Cadomian mountain range which contains granite rock zones formed millions of years ago. This natural resource enabled the granite industry to thrive in Louvigné du Désert forming an important part of the heritage [16].

There are no existing sensors on this trail. Some of the areas of interest are a significant distance apart and may require footfall sensors with the capability to communicate across long distances, either to Wide Area Networks (WAN) gateways or via cellular/telemetry.

#### 2.2.7. Taff Trail, Central Valleys, United Kingdom

The Taff Trail in the Central Valleys of Cardiff in Wales, UK, is a 55 mile trail between Cardiff and Brecon which takes in a mixture of riverside paths, railway paths and forest roads. It crosses Merthyr Tydfil County Borough, going through the spectacular Cefn Coed Viaduct and woodlands and runs to Pontsticill Reservoir in the Brecon Beacons where exceptional views of the highest mountains in the Brecon Beacons National Park can be enjoyed [17].

The Taff Trail has many entry and exit points and minimising counter redundancy will be very important; having too many sensors within a ‘complex’ area with numerous entry and exit points could lead to errors in footfall counting figures. There are existing thermal sensors on this trail, thus the integration of existing and future existing sensor data would be beneficial for analysis. Any newly introduced sensors will need careful consideration of location and placement so as not to cause ‘overlap’ on current detection areas.

#### 2.2.8. Serra d’Arga, Alto Minho, Portugal

The Serra d’Arga (Sacred Mountain) is a high granite plateau located between the municipalities of Viana do Castelo, Caminha and Ponte de Lima in Portugal. This iconic mountain region is famous for its natural and scenic qualities, as well as for its geological and archaeological interest. The Serra d’Arga trail is part of the European Union Natura 2000 Network, which aims to protect the long-term survival of Europe’s most valuable and threatened species and habitats [18].

The Sacred Mountain trail in Portugal is a circular trail with several points of interest. There are no existing sensors in place. The trail has few entry and exit points, increasing footfall count accuracy. Sensors placed at a common point of entry would be useful for gathering the amounts of trail users. Sensors could be used to monitoring the extent of the trail that is navigated by visitors or to monitor areas which are most commonly visited.

### 2.3. Sensor Usage on Trails

Outdoor environments are generally less hospitable to footfall sensors than indoor environments. Installing sensors outdoors can result in reduced count reliability due to environmental factors. Some sensors cannot be used outside as they are not appropriate, e.g., they may require resources such as mains power which may not be available in a rural location. Sensor technologies need to be frequently updated and older technologies can lose accuracy, as newer technology revisions are developed and released.

Footfall count data can be stored at any defined interval, providing that the sensor hardware can support the storage rate; that interval defines the granularity of the footfall data, e.g., minute-level or hourly. Fine data granularity may be desired to maximise the accuracy of footfall forecasts and mobility modelling, or even for real-time monitoring of occupation in certain pedestrian-rich areas. Outdoor footfall sensors are often battery-powered due to their environment, though as a consequence the frequency of data storage can negatively affect the devices battery life, owing to the energy required for data storage. If a fleet of sensors are installed and each requires regular battery changes, this can result in increased maintenance costs due to the physical labour required. Outdoor footfall data allows data gathering on a larger spatial scale than indoor footfall data. The latter is often implemented in a retail environment including shopping centres and supermarkets; their respective data is often focused on a single retail outlet but can occasionally be implemented in aggregate depending on the level of co-operation of ownership.

Capturing accurate levels of pedestrian traffic data can be problematic, a crucial factor in obtaining optimal counting results is physical sensor placement. Different arrangements of Thermal (non-vision) and PIR sensors are evaluated by [19] in an attempt to discover the maximum accuracy for their subject environment. The data is also compared with a ‘true’ manual count to establish a reference point for developing their calibration algorithm. In this situation, the thermal sensor was more effective at people detection; this is due to a thermal sensors’ ability to discriminate between living (warm) and inanimate (cold) objects based on their radiant heat signature. Although both technologies are commonly used, these sensor technologies have their limitations.

## 3. Footfall Sensor Technologies

This section introduces and discusses the different types of sensor technologies and considerations for optimal implementation. We investigate six different types of people-oriented footfall sensors; note some variants may not discriminate between people, bicycles or animals. Generally, if more discrimination is required, then a more technical (and therefore expensive) sensor type would be required. The six sensors are compared in Table 1, based on the key installation factors of power source and mounting method.

### 3.1. Camera/3D Based Visual Sensors

3D based visual sensors use cameras to analyse their surroundings and identify if a person has entered or exited a certain invisible threshold—which is usually a box or polygonal-shaped area. Computer Vision (CV) based systems offer the ability to accurately count visitors depending on lighting conditions and camera placement. These sensors are capable of recognising a passer-by’s direction of travel and can provide separate count values for people entering and exiting the camera’s Field of View (FoV). Vision sensors can be GDPR compliant, as the camera images can be processed and interpreted on the device and therefore not transmitted to a remote server, potentially compromising data security.

One of the challenges of vision-based systems is the ability to differentiate between different categories of objects; therefore, effectively differentiating people from animals can be challenging for a basic CV based system [20]. Some higher-end models can recognise individuals and achieve greater accuracy in counting people; other models do not differentiate individuals or objects and simply calibrate their count value by 50% to derive the one-in-one-out total. Another challenge with visual systems is their mounting requirements, effectively if the subject cannot *see* the camera, the camera cannot *see* the subject; this is known as Line-of-Sight (LoS). Unfortunately, mounting a camera in immediate view to passers-by may make it more susceptible to vandalism and critical damage. A Deep Learning (DL) based top-view people counting approach has been proposed in [21]. This technique allows the system to use a low profile, small footprint camera mounted on a ceiling or an archway, effectively out of view to the casual observer. Another top-view approach based on changes in video contrast is presented in [22]. Vision-based inputs have been combined with audio sensors in [23] to more effectively count people in non-ideal lighting conditions such as strong direct sunlight, or during the night. People counting approaches utilising CV based systems are evolving into crowd counting, with the desire to count and differentiate the demographics within a crowd with high accuracy. This is explored via a Multi-layer Convolutional Neural Network (CNN) in [24]. There can be significant power requirements and hardware overheads required to implement vision-based systems; this approach can require a lot of computation, particularly on the higher-end sensors. Internet or local network connectivity may also be required to transmit the information, or to send data for processing. The use of cameras in outdoor settings is particularly associated with a lack of privacy, and visible installation of these sensors may not compliment the atmosphere of an outdoor exploration trail.

### 3.2. Wi-Fi/BLE Device Monitor ‘Sniffer’

**Figure 1:**
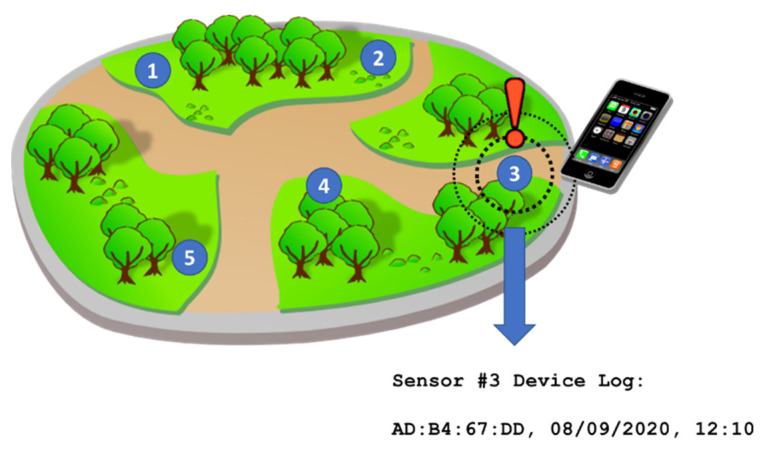
An example of Multiple WiFi Sensors (sensors 1-5) installed across a Forest Trail to track devices and hence track route of travel

**Figure 2:**
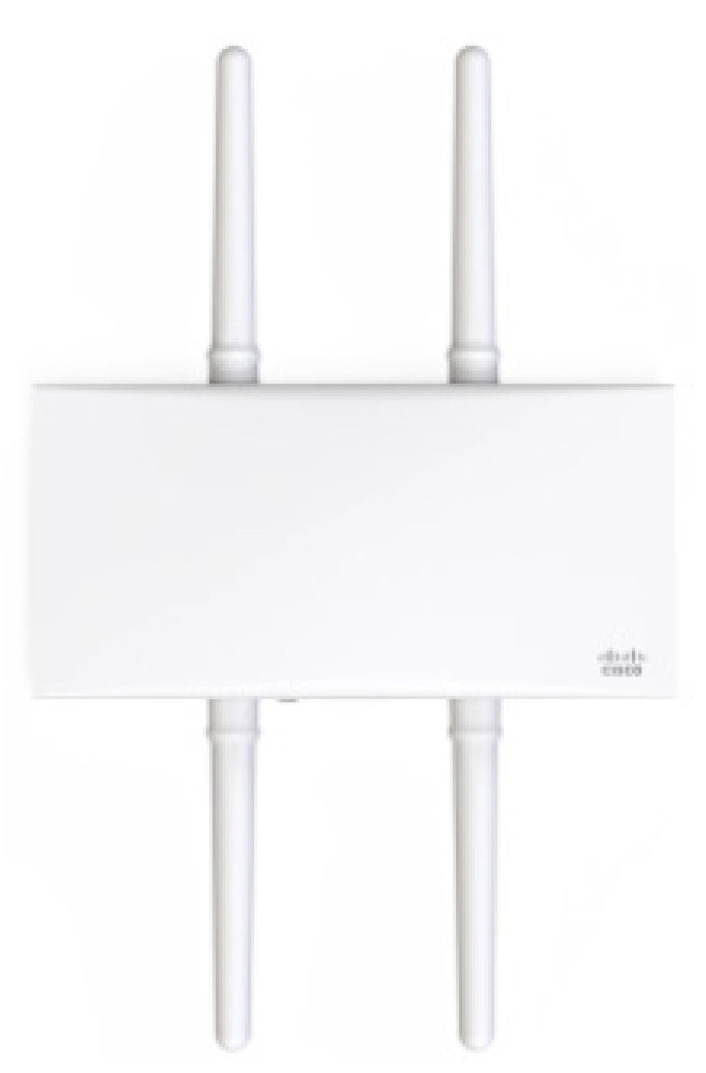
Cisco/Meraki ‘MR76′—Outdoor Wi-Fi & BLE Access Point.

This method of detection attempts to quantify WiFi and/or Bluetooth (BT) equipped devices within the proximity of the sensor. The majority of passers-by will carry modern Wi-Fi/BT equipped devices, such as a smartphone or a tablet [25]. Each device has a unique ‘signature’ called a Media Access Control Address (MAC Address) which is broadcast with each WiFi and/or BLE request signal; this signature is the basis of tracking individual devices. A device’s route of travel can be captured by distributing sensors throughout an area, as illustrated in Figure 1, and logging when the device has entered the sensors’ range. The sensors are very similar in appearance to a ubiquitous Wi-Fi access unit, but with additional communication radios, including BLE and 4G. The units are also toughened with waterproof enclosures for outdoor use. An example unit is shown in Figure 2.

Wi-Fi people counting can also be performed through the use of crowd-sourced Wi-Fi smartphone data [26]; this approach circumvents reliance on ‘exact location’ sensor data and increases count accuracy with additional smartphone data in aggregate.

A potential retrofit solution for existing Wi-Fi infrastructure is presented in [27] where Wi-Fi radio signal strength is modulated by the doppler effect; the perceived signal loss and passers-by can be processed by CNNs and interpreted as pedestrians walking past the sensor. Similarly, a device-free crowd counting approach is presented in [28] which also utilises the doppler effect on Wi-Fi signals. The differentiating factor here is that this approach only requires training once in each new environment. A Footfall count estimation approach has been proposed in [29] by analysis of mobile data access logs from nearby cellular towers; this approach circumvents MAC obfuscation and on-device privacy measures which are reducing the accuracy of ‘sniffer’ type devices. One of the potential drawbacks is that a trail user may carry multiple devices which could inflate the sensor’s count values.

Wi-Fi and/or BLE sensors can be placed out of view or mounted at elevation to reduce their susceptibility to vandalism. How often individuals visit a trail can be quantified and allows for analysis of returning visitors, and unique or new visitors can be differentiated. These sensors can also be powered by solar or mains power and require data access, they can be remotely updated and monitored. This remote monitoring can reduce maintenance costs compared to other sensors which require battery changes or manual data collection.

### 3.3. Radar (Doppler) Sensor

**Figure 3:**
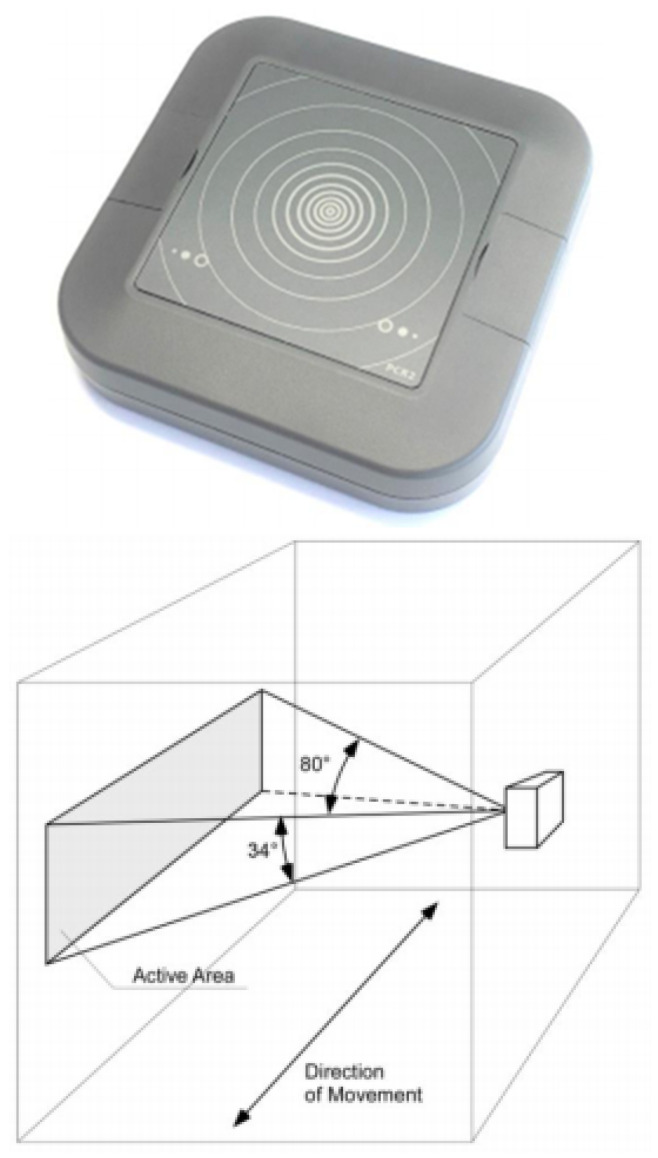
Parametric PCR2 Radar Sensor and FoV for Radar Sensor (PCR2 Equipment Manual).

Radar sensors, as shown in Figure 3, emit microwave frequencies and utilise the resultant frequency modulation effect to track an object that has entered its field of view [30]. As a person’s movement is tracked, their direction of travel can also be attained to produce separate ‘in’ and ‘out’ counts. The device can also ignore small disturbances such as birds that may enter the field of view. There is a wide range of radar sensors available, utilising different radio spectrums for detection; for example, the approach in [31] uses a short-range radar sensor to count individuals.

This type of sensor does not have vision or image sensors and as such are inherently private and GDPR compliant. Radar devices have a focused field of view that allows it to determine the visitor’s direction of travel to provide separate directional (in and out) counts for better analysis. Radar sensors can have a small device footprint, and benefit from a simple installation process. However, the sensor must be mounted within view of the passers-by which can make it susceptible to vandalism. These devices have a mounting specification, and the minimum mounting height will reduce the effectiveness of the sensor at counting smaller children. Battery power is generally unsuitable for this family of devices, as they have a high current requirement due to technology limitations; solar or mains power is most suitable for this implementation. LoRa and/or LoRaWAN data communication variants of these sensors are also available, which allow long-distance and low power communications without relying on cellular data or internet availability. In [32], radar sensors are used for moving crowd counting, with 97.5% accuracy in counting up to 4 people per square metre.

### 3.4. Pyro (Thermal) Sensor

**Figure 4:**
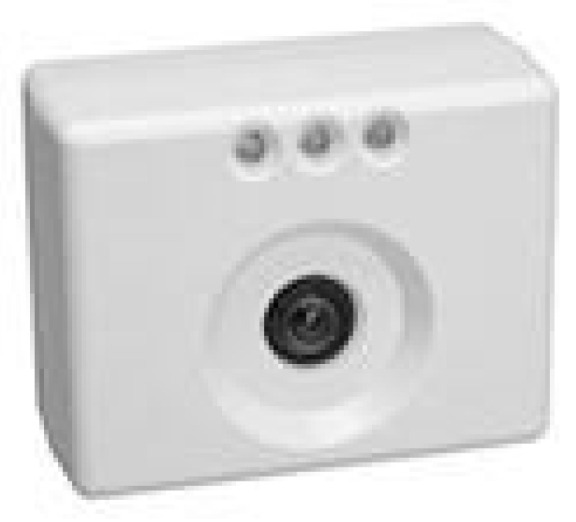
Pyro Thermal Sensor by FLIR.

Pyro sensors, such as the FLIR system’s thermal sensor in Figure 4 include some of the benefits of a vision-based system, but a key differentiator to other sensors types is that these sensors do not require specific lighting conditions, as an object’s radiant heat is visible to the sensor whether it is light or dark. These sensors can detect and differentiate objects within the field of view based on their radiant heat ‘signature’ [33]. Thermal sensors can derive the direction of travel and provide separate counts. They are also considerate of visitor privacy as the sensors cannot capture identifying features of pedestrians.

The authors of [34] use a low-resolution thermal camera in a crowd counting approach. Any detected heat signatures must be considered significant enough to be classed as a person, insignificant or interfering objects can be ignored; including small animals, fallout or nearby moving objects such as gates or barriers. The device must be mounted in-view and has a usable detection range of approximately 4 m.

### 3.5. Passive Infra-Red (PIR) Sensor

**Figure 5:**
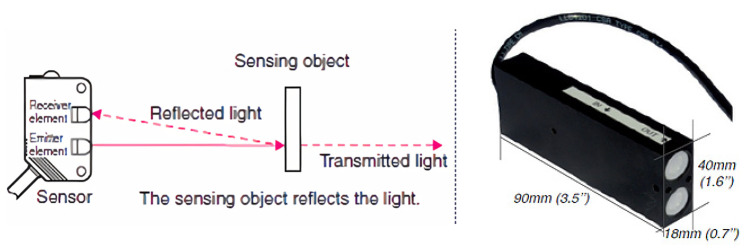
Eco Counter PIR sensor with a combined emitter and receiver.

Infra-Red sensors, shown in Figure 5, are used in a ‘break beam’ configuration for footfall detection. An invisible IR beam is focused on an IR receiver (sensor). If this beam between the emitter and receiver is broken, this would be interpreted as a person entering or exiting an area. Since the count is triggered by anything that ‘breaks’ the IR beam, count data may require aggressive calibration to increase counting accuracy. Dual IR sensors are required to detect the travel direction of passers-by [35,36].

Sensor configurations exist with both the emitter and receiver combined in one physical unit, minimising the device footprint. These sensors can be very discretely mounted and even routed into existing fixtures to minimise their appearance and reduce their susceptibility to vandalism. PIR break beam setups can be resilient to weather changes and consume very low power (typically < 100 mA) allowing them to run continuously for greater than a year on commercial alkaline batteries. These sensors are also available with cellular (3G/4G) communication and can be suitable for remote areas, especially when running on battery power. These sensors have a wide field of view, up to 180 degrees can be achieved with an appropriate lens. Careful sensor placement is required to avoid falsely triggering the sensor and avoiding strong direct sunlight.

### 3.6. Pressure Slab

**Figure 6:**
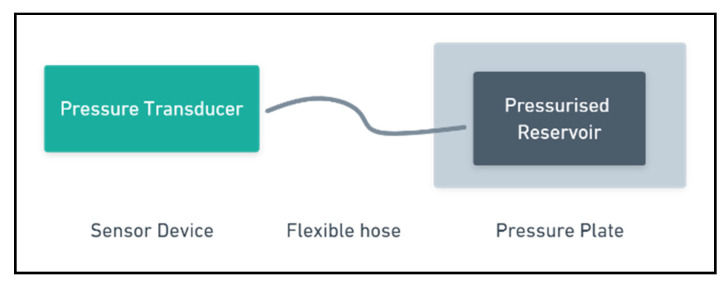
Block diagram of ‘Pressure Slab’ Components.

A pressure slab sensor consists of a gently pressurised flexible ‘reservoir’, sandwiched between two thin slabs of material. The slab element is installed underground, below the path to be monitored. The pressure in the reservoir is remotely monitored by a pressure transducer connected by a flexible hose. The transducer converts the pressure signal into a voltage. Changes in pressure will be reflected in the transducer’s output signals, these signals can then be processed and interpreted by a microcontroller as footfall [37]. In this arrangement (illustrated in Figure 6), a single pressure plate cannot determine the direction of travel, though multiple units can be combined to implement this functionality.

**Figure 7:**
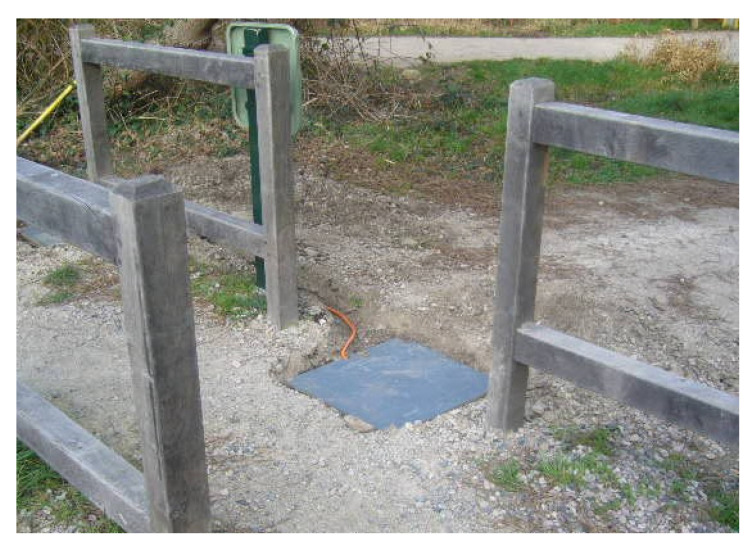
Eco Counter ‘SLAB’ installed at a pedestrian pinch point.

As the sensor is installed underground, it is effectively invisible in most environments. Due to the low maximum depth of burial, as demonstrated in Figure 7, this sensor may be susceptible to changes in environment such as soil erosion. The sensor can ignore small animals and minor disturbances as those events have a smaller seismic presence. A single slab with a transducer has a relatively small footprint, and multiple sensors can be installed to cover a larger area or to determine the direction of travel. The reservoir pressure can be calibrated on demand, to account for atmospheric variations. A drawback of this sensor approach is that continuous footfall with no gaps may not be effectively detected and counted.

It should be considered that many sensor equipment suppliers will offer hybrid options, for example—an inductive loop sensor and a PIR sensor can be combined to detect people and bicycles passing the same location, and count values can be differentiated in software for more effective data analysis.

## 4. Choice of Sensors

This section will compare some of the key differences of the sensor technologies and present a decision chart to assist decision making on which sensors will best suit a particular situation. The TrailGazers partners’ sensor choices are also discussed.

### 4.1. Guide to Appropriate Sensor Selection

Initially, trail managers should identify areas of their trail where sensors could be placed. Ideal sensor placement should monitor intentional footfall past that point. Placement considerations include avoiding higher activity areas with children playing and people loitering, or locations that visitors may pass several times during a visit.

**Figure 8:**
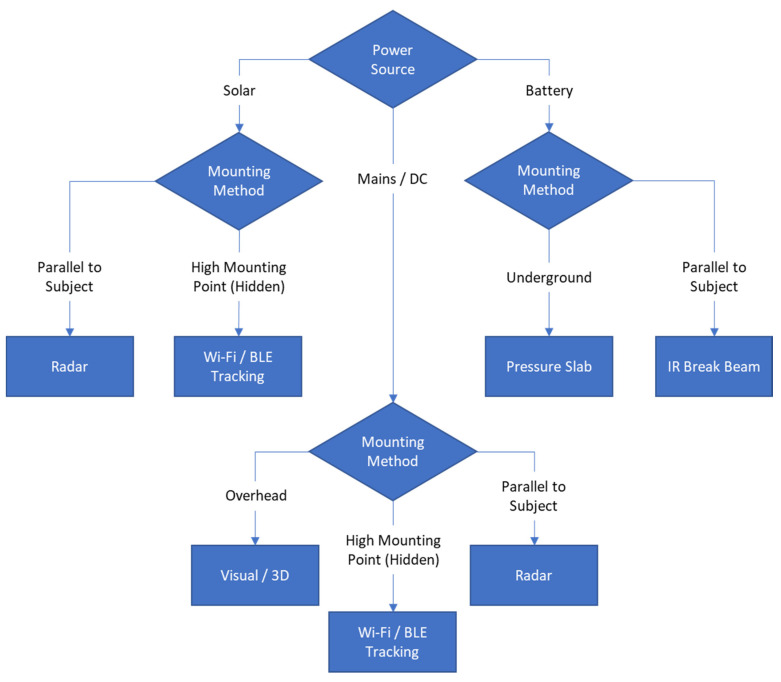
Sensor Technology Decision Tool (Developed from TGs sensor technology scoping study).

Sensors should ideally have automated data collection if budget permits. Manual uploading of data to a dashboard or analysis platform may be possible, but it will require physically visiting the sensors to collect the data regularly. The costs of doing so will have to be considered as part of maintenance. Sensors with manual data collection can be used as the detection mechanism for a scoping exercise to assess utilisation of a certain area of your trail without committing to a more expensive, automated data collection sensor. Figure 8 shows a decision chart that was developed from the TGs sensor technology scoping study. It considers requirements around mounting and power to aid trail managers’ decisions on which sensor technology to implement, for their situation.

Power source and mounting method are key factors in deciding which sensor technologies to implement; power supply requirements (e.g., Mains DC—if the device has high power requirements or is computationally heavy, or battery power if mains power is not available). The mounting method is the physical mounting height or specified mounting areas for maximum sensor effectiveness. Additional factors include usable detection range and data connectivity options (how the device will transmit the count data). A comparison of the sensor technologies discussed in Section 3 is presented in Table 1.

In summation, outdoor people and crowd counting sensor technologies continue to innovate, particularly around CV-based systems; the cost of computation is decreasing, and CV system frameworks are becoming more accurate with faster training algorithms. They also permit the desired ability to integrate with existing CCTV infrastructure, though dedicated purpose-built systems will offer the best performance. Edge computing is also revealing more opportunities for low power consumption vision-based systems [38]. An edge computing approach performs image processing on the device ‘in the field’; the video stream or images are not relayed via a wide area network (WAN) for processing like other approaches. This allows effective protection of anonymity, adhering to GDPR restrictions while allowing the powerful analytics and accuracy that CV can provide.

### 4.2. Sensor Technology Recommendations for Pilot Trails

A central component of the TrailGazers project is to develop sustainable trail management strategies in Ireland and across the EU Atlantic Area. Sensor recommendations are provided following an extensive review of the commercially available footfall technologies, trail topologies, dashboard data requirements and any issues with existing implementations. The scoping study of appropriate sensor technologies was performed through desk-based research and discussions with appropriate suppliers. This section discusses the partners’ technology options and rationale for implementation. The TrailGazers pilot trail partners have provided the following feedback on limitations of current sensors in place:

- Sensors are not always functional, readings can be erratic and cannot be corrected in the database, as the data is stored by another company. Occasionally this can be caused by changes in the environment surrounding the sensor, particularly over longer periods of time.
- Readings can be affected by nearby construction works. Reverberations of heavy machinery can affect the readings of acoustic/pressure sensors.
- Sensors are often vandalised, and expensive to repair.
- Sensors do not provide enough detail about the trail visitors e.g cannot differentiate between people, cars, bus and bicycles.
- Data requires manual collection, physical site visits.
- No feedback on fault conditions.
- Sensor data is not reliable and requires aggressive calibration (discovered through experimentation) to increase confidence.
- Multiple entry/exit points from trails complicate the accuracy of data presentation.
- No cellular (3G/4G) signal on-site—automated data collection is not suitable.

The partners’ feedback reinforces the need for new technologies to mitigate as many of the issues as possible. In one case, the company which supplied their original sensor installation has folded and will provide no future maintenance, repairs or support. Another partner has relatively accuracte technologies installed but the data is stored on a legacy system and cannot be externally accessed; the sensors also have gaps in the count data, which renders it unreliable. None of the currently installed sensor technologies on the trails support automatic data access. This feature is crucial for realising a combined trails dashboard. Existing sensor infrastructures, in this case, cannot be upgraded. Therefore, the best option for the trail partners is to replace their existing sensor infrastructures, which will be facilitated through the EU funded Trailgazers project. New sensor technologies will be installed on the 8 pilot trails to complement their existing sensor arrangements, or as an element of a new trail management strategy. The partners’ feedback will be carefully considered when providing their personalised recommendations. Generally, improvements in detection accuracy can be achieved with a more appropriate sensor type or through the combined use of multiple sensors to suit the trail topology. Error range data for the sensors is not generally available, as this can greatly vary depending on environment and installation arrangements. Sensor suppliers also provide installation to maximise detection accuracy in the areas defined by the trail management.

#### 4.2.1. Inch Levels, Donegal, Ireland

Inch Levels, Donegal, Ireland is a complicated trail from an entry and exit point of view and it may be difficult to achieve optimal sensor placement. Device-level Wi-Fi and/or BLE tracking could provide the best opportunity for counting trail visitors, as it is important to keep the data as accurate as possible. Wi-Fi and/or BLE sniffing sensors can be distributed throughout the trail and be utilised in aggregate to track the trail visitor’s journey. However, Wi-Fi and/or BLE sensors need a mains power supply and internet connectivity, which could potentially be an issue on this rural trail. An alternative technology choice is a bi-directional variant of PIR Break Beam sensors, which could be deployed to gather direction of travel and enhance qualitative data findings. Visitors can enter or exit at different points of the trail and analysing the balance of mobility between those locations could provide valuable insights. There are PIR sensor varieties which use cellular communication (if signal is available) and utilise battery power, with a life of up to two years; this could greatly reduce any maintenance costs.

#### 4.2.2. Knocknarea/Killspugbrone Loop, Sligo, Ireland

Knocknarea/Killspugbrone Loop, Sligo, Ireland—Sligo County Council trail management have expressed an interest in counting and differentiating between bicycles, adults and children. This could be achieved with a 3D Vision camera-based system, effectively differentiating and counting these different groups. The common caveat applies, mains power and internet connectivity are required but this option is also the most expensive. Alternatively, PIR break beam sensors placed in a ‘bicycles only’ choke point and/or entry area will increase the accuracy of the bicycle count. PIR sensors will count anything that breaks the invisible beam. People can similarly be forced through a ‘choke point’ that does not allow bicycles, such as a turnstile. Differentiation between passing adults and children will require a more intelligent sensor such as Radar or Pyro at an effective mounting location. Cellular and/or internet communications will reduce or remove the need for visiting the sensors for maintenance or data retrieval.

#### 4.2.3. Vía Verde Del Plazaola, Navarra, Spain

The Vía Verde Del Plazaola Trail, Navarra, Spain, is long and has few entry/exit points, though the level of traversal of the trail is not known. This analysis could be achieved through Wi-Fi and/or BLE device tracking, as these sensors can be used to derive a user’s exact journey, including dwell times and overall visitation time, though mains electricity and cellular signal may not be available throughout the trail. An alternative option that could be more suitable is radar sensors complemented by solar power or IR Break Beam sensors with battery. If it is important to accurately track bicycles entering and exiting the trail, then new choke points at the trail entries and exits could be installed to force bicycles to be counted individually from people using lower situated technology sensors.

#### 4.2.4. La Caldera De Taburiente, La Palma, Isla Canarias, Spain

The La Caldera De Taburiente Trail, La Palma, Isla Canarias, Spain, has expressed a desire to quantify adults and children. If separate counts are required, a Vision-based sensor system can differentiate between the two different categories, though mains power and internet connectivity are required. A combined count could be achieved by using PIR break beam sensors, at appropriate placement. Break Beam sensors should ideally be mounted 70 cm or greater above ground level, therefore smaller children will not be detected. There are variants of these sensors with cellular communications (2G or 3G signal depending) and battery power.

#### 4.2.5. Sete Vales Suspensos, Algarve, Portugal

The Sete Vales Suspensos Trail, Algarve, Portugal, has no existing sensors and there are no areas currently being monitored on the trail. The trail is long and has many points of entry and exit. There is a local bus to facilitate travel between the legs of the trail, so an individual may not experience the trail completely, or even in order. This is an initial footfall scoping exercise, so the detection strategy is aimed at discovering the popularity of the trail legs. Individual trail users could be tracked using WiFi and/or BLE (Type 2) sniffing sensors, and distribution of these sensors throughout the trail legs could monitor which legs the visitor will experience. A limitation is that WiFi and/or BLE sensors need a mains power supply and data connectivity, which could cause problems with installation, budget is another consideration, as there are many areas which should be monitored and could be expensive. Otherwise, Bi-directional variant of PIR Break Beam sensors could be employed to gather direction of travel and allow more flexibility in detection. This will also help enhance any qualitative data findings, as mentioned previously. There are varieties which use cellular communication (if signal is available) and can last two years or more on alkaline batteries. Purchase costs can be reduced by installing manual data collection variants and allow the purchase of a greater quantity of sensors.

#### 4.2.6. Chemin De Mémoires, Ille-Et-Vilaine, France

The Chemin De Mémoires Trail, Ille-Et-Vilaine, France, areas of interest are distributed throughout a town, so entry and exit levels are very complex. Wi-Fi/BLE Tracking allows the ability to track the time a visitor spends at a certain location as well as monitoring their journey around those different points of interest. If there is no mains power available, IR break beam is an alternative option. A distributed count of visitors at each area of interest can be achieved by installing affordable PIR sensors, powered by batteries for long periods and use 3G or 4G for automated data collection. These sensors are designed to be triggered when the invisible beam is broken and requires careful consideration of sensor placement, as anything that breaks the beam will trigger the sensor.

#### 4.2.7. Taff Trail, Central Valleys, United Kingdom

Merthyr Tydfil County Borough Council have expressed an interest in quantifying people on horseback as well as individual people; this could be achieved using PIR sensors mounted at an appropriate height, above the height of people. The potential to differentiate footfall counts as adults and children will require a more complicated sensor solution. A vision-based system, such as a 3D stereoscopic camera (Type 1) could be used to accurately differentiate between all the desired demographics but will be more costly than other sensors. Existing sensors utilise mains power and ethernet WAN connectivity, new sensor systems can also utilise this for automatic data communication.

#### 4.2.8. Serra d’Arga, Alto Minho, Portugal

For the Serra d’Arga, trail managers have expressed an interest in counting adults, children and bicycles. If separate category counts are required, a vision-based camera system could effectively differentiate and count these groups, but it would require mains power and internet connectivity. A LoRa communication network is under installation in this area and could allow LoRa communication variants of radar sensors that could also differentiate between these groups. Finally, PIR break beam sensors could be implemented—these sensors will count anything that breaks the invisible beam. Therefore, placing these sensors in a ‘bicycles only’ choke point/entry area will increase the accuracy of the bicycle count. In addition, cellular and/or internet communications, if applicable, could reduce or remove the need to visit the sensors for data retrieval. The next steps for trail management are to consult the technology decision chart devised during the TrailGazers project (Figure 8) and ensure their chosen technology will suit their installation situations, throughout the trail. Once the technology has been decided, the partner will proceed to procurement of the sensors to find appropriate suppliers.

In summation, each trail partner must procure the most appropriate sensors to suit their respective trails and trail management objectives. An open-source trail monitoring dashboard will be developed as part of the project, which will link to the captured sensor data and provide data insights on trail usage patterns, as well as offering predictions for future trail visitor numbers to aid decision-making. Developing this platform will require automated online access to the data from the sensors via an Application Programming Interface (API) or by accessing the service provider’s database. This is a feature the project’s sensors must possess.

The dashboard platform will be populated when the trail sites are active. There are many planned uses of trail data throughout the TrailGazers project, including:

- Early identification of future trends—e.g., cyclo-tourism or accommodation of regular walking groups/swarms. Previously, some trails have been managed on how they are currently utilised. Prediction of trends will help produce future-proof management plans.
- Targeted area monitoring will help identify localised areas of the trails to develop strategies to minimise soil erosion and mitigate geomorphic effects [39]. Managerial decisions can contribute towards soil erosion, due to increased footfall or trail usage which has a detrimental, and lasting effect on trails [40].
- Footfall data will be used to develop management strategies to minimise off-trail use, which can have a damaging effect on nature around the established trails. Off-trail exploration can also indirectly create unmanaged informal trails [41].
- Data will be used to monitor visitor behaviour, which routes are most commonly used, and provide insights on how to enhance the visitor experience such as: maintaining vegetation, providing more safety measures, and justifying the introduction of outdoor eating facilities.

## 5. Conclusions

This paper has presented the key research outcomes from a scoping study into footfall sensor technology options, with an emphasis on the TrailGazers project—which is funded by the EU Interreg Vb Atlantic Area programme [42]. The trail partners’ individual recommendations have been presented, along with important considerations linked to sensor technology choices. The trail partners’ final technology decisions will also depend on their identified Key Performance Indicators whcih will make up a large part of trail management plans around, for example, aiding the protection of trails that are of cultural, environmental and historical importance. There is also a need to weigh up the cost of the technologies against their requied specifications, whilst bearing in mind that b udget limitations may impact on the quantity and type of sensors that can be procured and implemented. Maintenance and licensing costs are an additional consideration, particularly for manual data collection-based sensors, as they will require physical visits to collect the data. Otherwise, maintenance costs are relatively low after installation. A key decision-making tool to aid sensor technology choices within tourist trails located in various regions of the Atlantic Area of Europe has been developed and presented in this paper. There is potential for wider application. For example. given the significant role of tourism and tourism attraction systems on the island of Ireland and beyond, in Europe, there will be ongoing needs to monitor tourism foorfall and develop trail management plans for trail sites; many of which are located in areas of outstanding natural beauty and need considerate management to conserve them. This tool also has wider application for decision making linked to any outdoor footfall monitoring scenario.

## Figures and Tables

**Table 1:** Sensor Overview and Comparison.

Discussion Section	Sensor Type	Detection Range	Mounting Requirements	Power Requirements	Data Connectivity Requirements
**Section 3.1**	**Visual/3D**	Lens and mounting position-dependent.	Overhead.	Mains DC	Ethernet/WiFi/GSM
**Section 3.2**	**Wi-Fi/BLE Sniffer**	Approximately 20 m radius.	3 m or above.	Mains DC	Ethernet/WiFi/GSM
**Section 3.3**	**Radar**	6 m ‘conical’ area.	Parallel (~1.2 m from floor).	Solar/Mains DC	Model dependent
**Section 3.4**	**Pyro Thermal**	4 m ‘conical’ area.	Above 70 cm.	Solar/Mains DC	Ethernet/WiFi/GSM
**Section 3.5**	**PIR Sensor**	10 m linear beam.	Above 70 cm.	Battery DC	None
**Section 3.6**	**Pressure Slab**	Equal to size of slab.	Underground.	Battery DC	None
